# Supplementary material for: Cloning and Characterization of Chitin Deacetylase from Euphausia superba
Source: Int J Mol Sci. 2024 Feb 8;25(4):2075. doi: 10.3390/ijms25042075 (PMC10889134; doi:10.3390/ijms25042075)
Supplement: Supplementary file 1 [file ijms-25-02075-s001.zip › ijms-2835511-supplementary.pdf]

Supplemental material

Supplemental Figure:

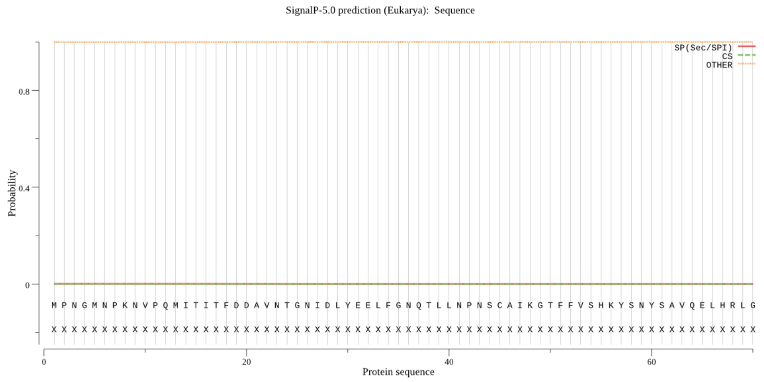

Figure S1. Signal peptide analysis.

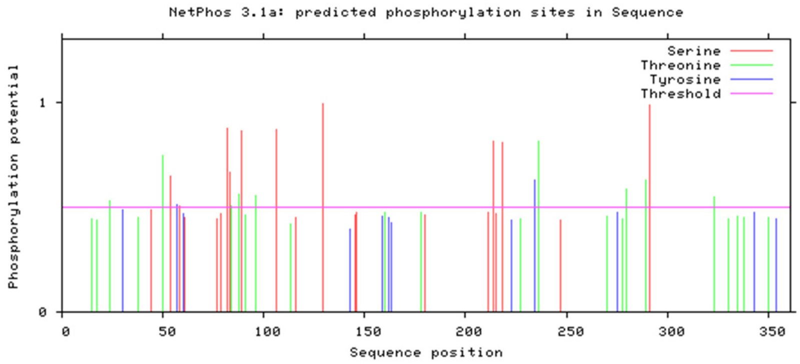

Figure S2. Phosphorylation site analysis.

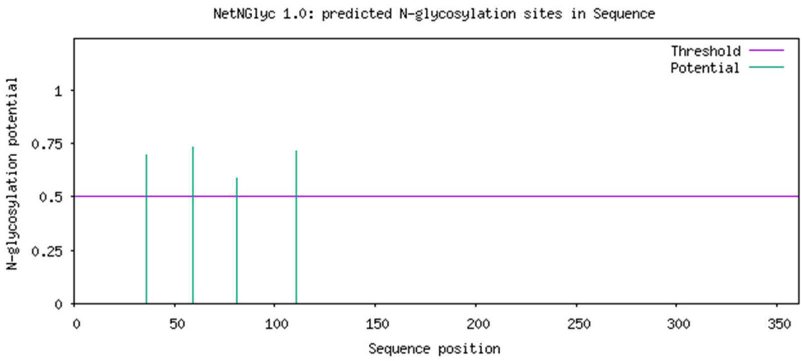

Figure S3. Glycosylation site analysis.

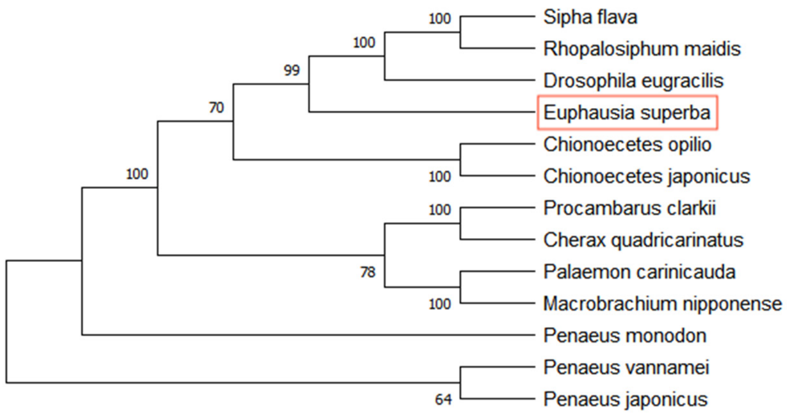

Figure S4. The phylogenetic tree constructed according to the sequences of *Es*CDA and other CDA. *Es*CDA researched in this study is in red box.

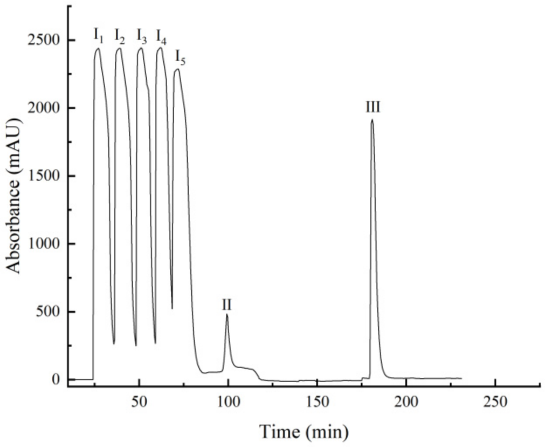

Figure S5. Ion exchange chromatography absorption peak.

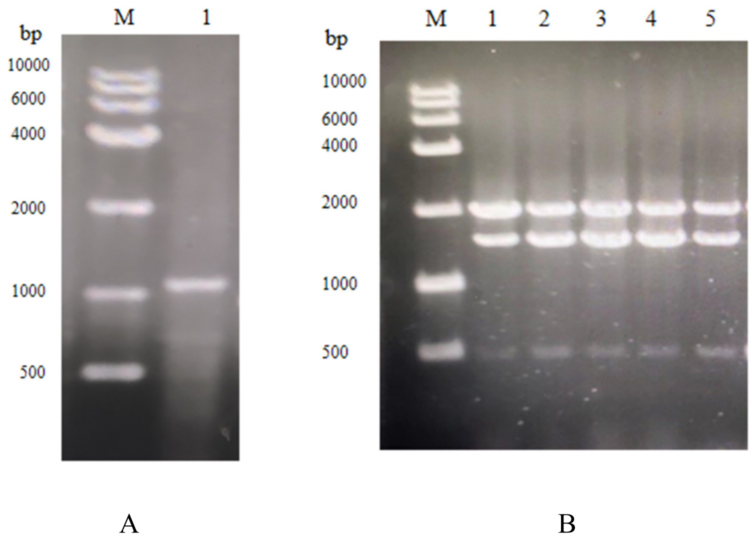

Figure S6. Results of agarose gel electrophoresis. A: Amplification analysis of the CDA genes.

Lane M: DNA marker; Lane 1: PCR amplification product. B: Colony PCR products for verification of DH5α transformants of *Es*CDA. Lane M: DNA marker; Lane 1-5: Positive colony PCR products of DH5α transformants of *Es*CDA, including *Es*CDA genes and partial expression vector (pPIC9K universal primers: 5'AOX I and 3'AOX I; 2000bp: AOX 1 gene in the GS115 genome; 1600bp: target gene+pPIC9K part gene; 500bp: dimer formed during the PCR process)

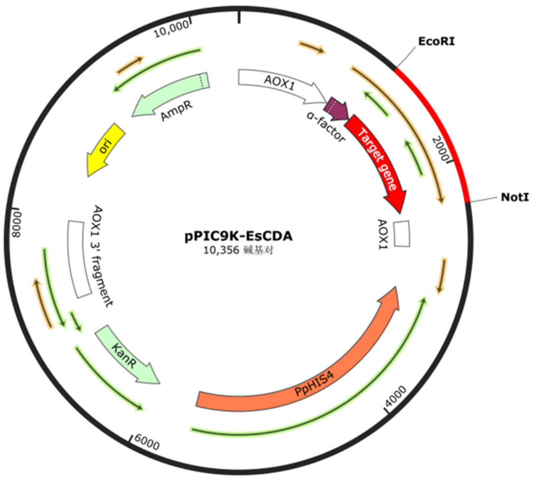

Figure S7. Construction of recombinant plasmid pPIC9K-*Es*CDA.

Supplemental Table:

Table S1. The biochemical properties of different chitin deacetylases.

Strain	Optimum temperature / °C	Optimum pH	Molecular Weight / kDa	Reference
Absidia coerulea	50	5	75	19
Saccharomyces cerevisiae	50	8	43	21
Scopulariopsis brevicaulis	55	7.5	55	22
Rhizopus circinans	37	6	75	23
Mortierella sp. DY-52	60	5.5	50	24
Aspergillus nidulans	50	7	27	36
Metarhizium anisopliae	-	8.9	70	38
Saccharomyces cerevisiae	50	7	34	45
Coprinopsis cinerea	70	7.0	28	46

Table S2. Mutation PCR reaction system.

Reagent	Sample volume (μL)
Q5 Hot Start High-Fidelity DNA Polymerase	12.5
forward primer	1
reverse primer	1
template DNA	1
nuclease-free water	9.5

Table S3. Primers used in mutagenesis.

Mutation	Sequence (5′-3′)
F18G-F	TACCATTACCGGTGACGACGCCG
F18G-R	ATCATTTGGGGCACGTTC
R121D-F	TATTGGTATGGATGCTCCATTTAACAGAG
R121D-R	ATGGATTGGTCAGTAATG
F124G-F	GAGAGCTCCAGGTAACAGAGTTGG
F124G-R	ATACCAATAATGGATTGGTC
Q246H-F	TTTGTTTTTTCATTCTGCTTTTTTGAAG
Q246H-R	CCAAGTGGTGCTCTATTAG
